# Enhancement of exfoliating efficacy of L-carnitine with ion-pair method monitored by nuclear magnetic resonance spectroscopy

**DOI:** 10.1038/s41598-019-49818-2

**Published:** 2019-09-18

**Authors:** Sohyun In, Naeun Yook, Jin-Hyun Kim, Munju Shin, Suryeon Tak, Jeong Hoon Jeon, Byungjun Ahn, Sun-Gyoo Park, Cheon-Koo Lee, Nae-Gyu Kang

**Affiliations:** R&D Campus, LG Household & Health Care, 70, Magokjungang 10-ro, Gangseo-gu Seoul, Republic of Korea

**Keywords:** Drug delivery, Mechanism of action

## Abstract

Carnitine (CAR), an amino acid derivative, has great potential as a facial exfoliating agent owing to its calcium chelating property under weakly acidic or neutral conditions. However, its application is limited by its poor transdermal penetration. To optimise its exfoliation efficacy with minimal concentration, we propose the ion-pair method. The ionic interaction between CAR and a zwitterionic substance was successfully monitored by measuring conductivity. The alterations of penetration and exfoliation efficacy for CAR addition to different types of counter ions were investigated *in vitro* and *in vivo*. We found that hydrogenated soya phosphatidylcholine (HSC), an amphiphilic counter ion, significantly increases the stratum corneum penetration and exfoliation efficacy of CAR. The changes of the CAR-HSC ionic interaction in the presence of calcium ions were also investigated by ^1^H nuclear magnetic resonance (NMR) spectroscopy. The NMR spectra for amino groups of CAR first decreased with HSC and then gradually recovered and shifted as calcium ions were added. From the results, a noble exfoliating complex of CAR with high exfoliation efficacy could be proposed. Moreover, the results demonstrate that NMR spectroscopy is useful to obtain direct experimental evidence of the molecular dynamics simulations of the alteration of an exfoliating complex as it penetrates.

## Introduction

The skin forms an effective barrier between an organism and its environment, preventing the invasion of pathogens and defending against chemical and physical assaults. The stratum corneum (SC) is the outmost layer of skin and the main barrier. The continuous production of SC is balanced by its desquamation^1,2^.

The decrease in the rate of desquamation caused by abnormal skin conditions, such as ageing and UV stress, is either a primary or principal associated reason for dermatologic disorders^3–5^. Many skin problems can be alleviated by treating the abnormal desquamation of SC; thus, the cosmetic and dermatologic therapeutic industries have great interest in exfoliating methods^3,6^.

Among these methods, the chemical exfoliating method has withstood the test of time, with superficial peels, in particular, remaining a popular tool. Moreover, alpha hydroxyacids (AHAs) are now widely used exfoliating agents^7–9^. However, AHAs have side effects, such as itchiness, redness, sting, and burning, arising from their low-pH acting condition and keratolysis properties^10–13^.

**Figure 1:**
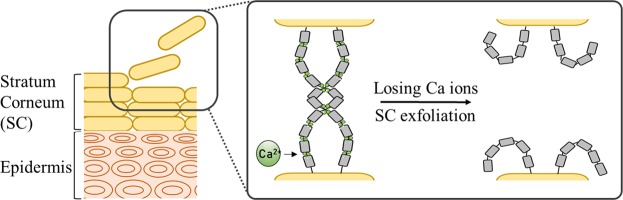
Schematic representation of the alteration of structure formation and interaction of desmosomes dependent on the concentration of Ca ions. Each desmosome in SC that is composed of five domains is represented by five repeated rectangles and Ca ions and corneocytes are represented as green circles and yellow ovals, respectively. A decrease in Ca ions in SC layer makes desmosomes to lose their *cis*-dimerization and *trans*-interaction, and weakens the adhesion of adjacent corneocytes causing SC exfoliation.

To overcome the side effects of AHAs, there have been many attempts to develop novel exfoliating agents^13–15^. In a previous study, Ahn *et al*. showed that the hydroxyl group of amino acids or amino acid derivatives, especially Carnitine (CAR), could be a novel exfoliating agent^16^. Ca plays an important role in the adhesion of adjacent corneocytes, and so, a decrease in the Ca ions in skin reduces the cohesion between corneocytes^17–21^. The effect of decrease in the concentration of Ca ions in the SC layers on the structure formation and interactions of the desmosome, a kind of cadherin that exists in tissues and is mainly involved in cell-cell adhesion, is represented in Fig. 1^17,21^. CAR effectively chelates Ca ions at weakly acidic or neutral pH; therefore, CAR could exfoliates SC without any adverse effects arising from low pH or keratolysis properties.

Although CAR has obvious benefits as a noble exfoliating agent, its application is limited by its poor transdermal penetration properties^22,23^. Even if amino acid derivatives are widely known to be safe relative to other chemicals, increasing the quantity of CAR to overcome its low penetration may cause safety issues. Several previous studies have mentioned the alteration effect of cellular processes, such as muscle-cell contraction, blood coagulation, and neurotransmission, caused by Ca chelation^17,18,24–27^ and the long-term effects of such cellular changes occurring in the dermal or deeper regions have been rarely studied. We intended to avoid the unnecessary and ambiguous safety issue by confirming that CAR, under our usage conditions, does not penetrate deeper than the dermal layers. Therefore, to optimise the efficacy of this amino acid derivative with minimal dose, the improvement of its penetration depth is vital in clinical facial applications.

In recent years, various skin-penetration enhancement strategies using penetration enhancers, vehicles, or derivatisation have been proposed. The merits of CAR as a novel exfoliating agent arise from its structural property to chelate Ca ions effectively. Also, even if considering the unexplored safety issue of CAR regarding deeper penetration mentioned above, CAR holds a dominant position compared with its newly synthesized derivatives. In developing a penetration enhancement strategy, we considered how effectively it could increase the penetration of CAR without losing its advantages. We found that this could be best addressed using the ion-pair method. Furthermore, this is a simpler and cheaper method without the use of specific devices or modification of the substance structure but with a proper counter ion^22,23,28^. Therefore, we used the ion-pair method in this study.

Penetration-enhancing studies for ionic substrates have thus far not studied whether this skin-transport enhancement method, or the ion-pair method, can be applied with CAR, an ionic exfoliating agent, to enhance penetration between SC layers. Further, the previous studies did not consider the acting sites of exfoliation, whether in the deeper dermal layers or not, or the possibility of side effects from this process, which are still under debate.

We attempted to evaluate the alteration of the amount and penetration depth of CAR with the ion-pair method by *in vitro* and *in vivo* tests. We also explored the disruption of the CAR-counter ion interaction in the presence of calcium ions by using nuclear magnetic resonance (NMR) to investigate whether the ion-pair complex of CAR works properly after skin penetration. Finally, we investigated the enhancement of exfoliation efficacy caused by the penetration enhancement of CAR with the ion-pair method.

**Figure 2:**
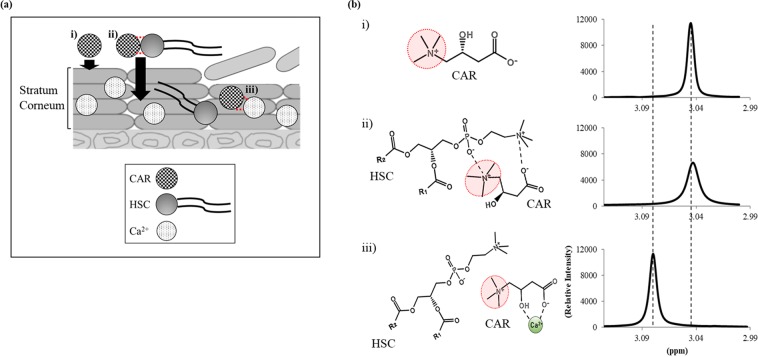
Proposed scheme of the effect of the enhanced penetration of CAR on its exfoliation efficacy and assessment of alteration of interactions of CAR in this process by NMR. Summary of the proposed mechanism of increase in the exfoliation efficacy of CAR through penetration enhancement method using HSC (**a**). CAR, HSC and Ca ions are represented as check pattern, gradation pattern and dotted circles, respectively. Corneocytes are represented as ovals. Interactions between CAR and HSC or CAR and Ca ions are drawn as red dashed lines. Schematic representation of alteration of interactions of CAR and ^1^H NMR spectra of amino groups in CAR at each case (**b**); in the case of CAR only exists (i), CAR makes electrostatic interactions with HSC (ii) and CAR breaks up interactions with HSC and chelates Ca ions (iii).

Figure 2 presents the schematic representation of the proposed mechanism for the use of ion pairing to enhance skin penetration and increase the exfoliation efficacy of CAR, as assessed by NMR spectroscopy. This comprehensive study not only enables dermatologists to understand the effect of noble amino-acid-derived exfoliating-agent treatment on abnormal skin, but also demonstrates how NMR spectroscopy can be used as an effective evaluation method to monitor molecular interactions in a complex system.

## Results and Discussion

### Conductivity experiments for monitoring electrostatic interaction of CAR

**Figure 3:**
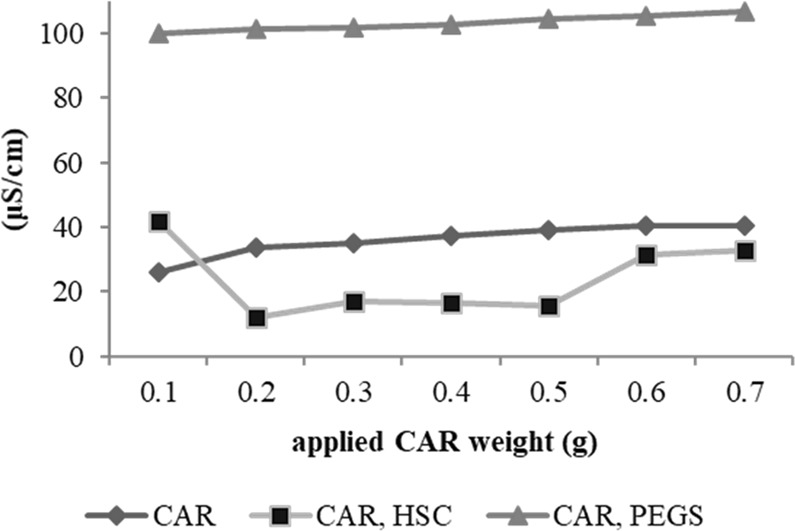
Conductivity changes in CAR mixed with zwitterionic or nonionic substance. Conductivity for carnitine (CAR) and hydrogenated soya phosphatidylcholine (HSC) mixture and for CAR and PEG-40 stearate(PEGS) mixture compared with the values for CAR only. Each sample is measured at 25 °C by adding CAR to 100 mL of 0.02 M HSC or PEGS solutions. All the values of standard error for CAR only and mixture with HSC or PEGS are less than 0.7, 0.2 and 1.0, respectively.

Carnitine (CAR) exists in a zwitterionic form under weakly acidic or neutral conditions. We investigated whether a zwitterionic substance could effectively form an ion pair with CAR, in contrast to nonionic substances. The alteration of conductivity on adding CAR to 0.02 mol/kg hydrogenated soya phosphatidylcholine (HSC) or PEG-40 stearate (PEGS) solution was investigated using a conductivity meter, and the results are shown in Fig. 3. The conductivity decreased as CAR was added to HSC, with the maximum decrease occurring in a CAR weight range of 0.2–0.5 g. In contrast, the conductivity did not decrease at any point as CAR was added to PEGS. For HSC, the weight of CAR with a 1:1 molar ratio is approximately 0.32 g. The results not only revealed that HSC had electrostatic interactions with CAR while PEGS did not, but also implied that conductivity significantly decreased around a molar ratio of 1:1 between CAR and HSC.

### *In vitro* and *in vivo* study of penetration changes of CAR induced by counter ions

We designed an experiment to analyse the ion-paring effect of CAR with zwitterions of different molecular structures. Overcoming the penetration barrier of SC for zwitterionic substances requires not only a decrease in polarity but also an increase in lipophilicity. For verification, we prepared samples combined with betaine (BT), polyquaternium-51 (PQ), or HSC as hydrophilic small molecular, hydrophilic polymeric molecular, and amphiphilic molecular zwitterionic counter ions, respectively. Each sample was prepared with 4 wt% CAR and the same molar ratio with BT, PQ, and HSC.

**Figure 4:**
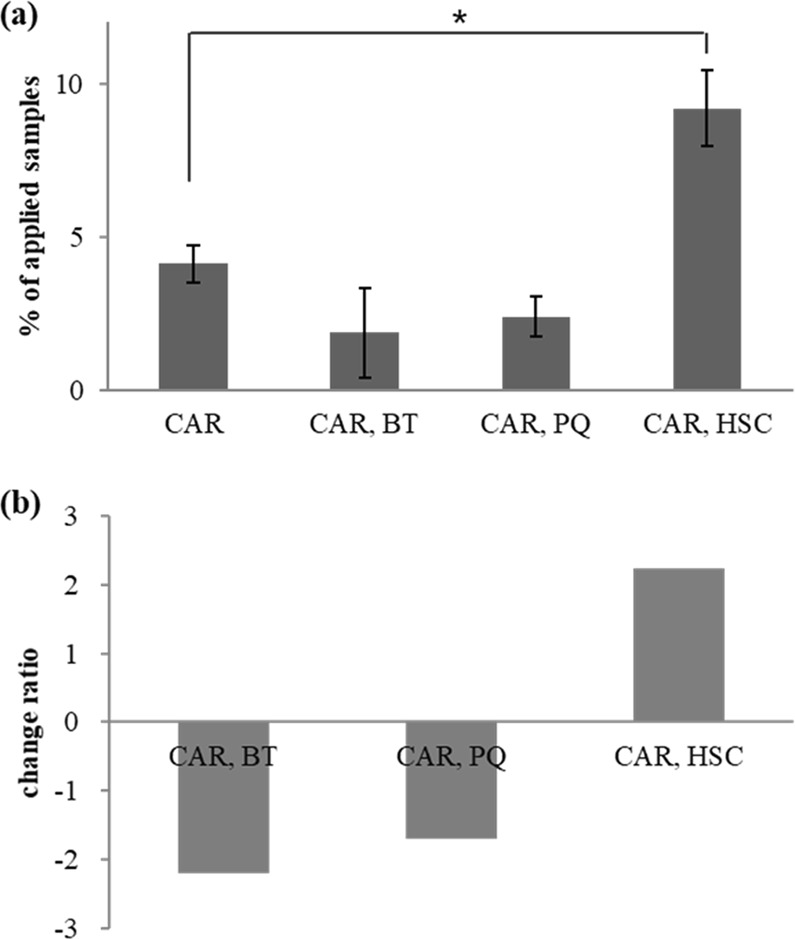
Alteration of percutaneous penetration for CAR mixed with BT, HQ or HSC. The penetration amount of 4 wt% CAR with and without same molar ratio of counter ions, BT, PQ or HSC into SC layers are represented as percentage of applied samples (**a**) and decrease or Increase in CAR penetration are represented as change ratio (**b**). Data are means and bars represent the standard error of them. *p < 0.05; Student’s *t*-test.

Table 1 summarises the effect of three different counter ions on the CAR penetration in SC. The CAR penetration was significantly increased with HSC relative to the control, but it decreased with BT or PQ. To quantify the variations in the penetration characteristics of CAR in SC, the change ratios were calculated, as shown in Fig. 4. HSC resulted in an approximately 2.2-fold increase in the CAR penetration. This result implies that the skin penetration of CAR could be changed by ion paring and that providing lipophilicity to CAR with an amphiphilic counter ion is important.

**Table 1:** Percutaneous penetration of CAR *in vitro* tests.

Percentage of applied product	CAR	CAR, BT	CAR, PQ	CAR, HSC
SC	4.12 ± 0.60	1.87 ± 1.46	2.38 ± 0.65	9.19 ± 1.24
Other epidermal layers	5.01 ± 2.00	2.38 ± 1.77	4.17 ± 2.46	14.52 ± 5.70
Dermal layers	N.D.	N.D.	N.D.	N.D.
Effluent fraction	N.D.	N.D.	N.D.	N.D.

Penetration alteration of 4 wt% CAR with same molar ratio of three different types of counter ions, betaine (BT), polyquaternium-51 (PQ) or HSC. The *p*-value for each sample vs. control by Student’s *t*-test is less than 0.5.

Another remarkable point is the penetration depth of CAR. While glycolic acid and lactic acid, which are representative AHAs, penetrated deeper than the epidermal layers, CAR penetrated only the SC and epidermal layers, even though the penetration efficacy was enhanced by ion pairing with HSC (Table 2). The penetration depth is an important factor related with the efficacy and safety of exfoliation. This result suggests that appropriate doses of CAR and its penetration enhancer, HSC, enables the penetration of CAR only into SC and the epidermal layers, minimising the possibility of safety issues.

**Table 2:** Percutaneous penetration of CAR *in vitro* tests compared with glycolic acid and lactic acid.

Percentage of applided product	4% CAR	4% glycolic acid	4% lactic acid
SC	4.12 ± 0.60	14.48 ± 7.01	10.60 ± 6.01
Other epidermal layers	5.01 ± 2.00	4.75 ± 1.06	10.05 ± 4.28
Dermal layers	N.D.	13.05 ± 9.68	11.80 ± 7.04
**Effluent fraction**			

Percutaneous penetration of CAR, glycolic acid and lactic acid. All data are mean and ±standard deviation. N.D. means not detectable.

**Figure 5:**
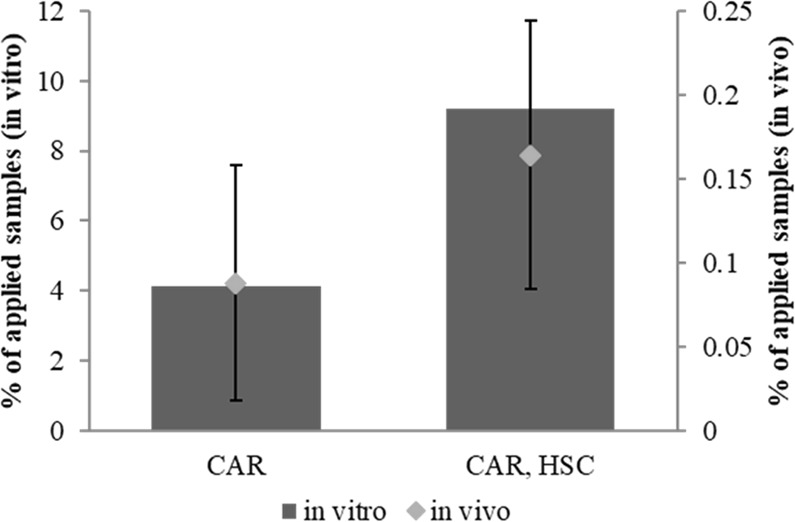
Alteration tendency for CAR penetration into SC layers induced by HSC *in vitro* and *in vivo* tests. Bar graphs and diamond-shaped dots represent *in vitro* and *in vivo* data for the penetration amount of CAR into the SC layer, respectively. Data are means and bars represent the standard deviation.

We also conducted an *in vivo* penetration study to confirm whether the ion-paring system of CAR and HSC works well in real skin. Each sample was prepared by increasing the quantity of CAR up to 10 wt% under consideration of the detection limit of HPLC. As indicated in Table 3, the percutaneous absorption of CAR in SC was approximately 0.08% of the applied sample and was increased by a factor of approximately 1.8 with HSC. This *in vivo* examination reveals that the penetration enhancement by HSC is well operated in real skin, with a tendency similar to that in the vitro test (in Fig. 5).

**Table 3:** Percutaneous penetration of CAR *in vivo* tests.

Percentage of applied product	CAR	CAR, HSC
SC	0.08 ± 0.07	0.16 ± 0.08

Penetration alteration of 10 wt% CAR with HSC. Data are mean and ± standard deviation. The *p*-value by Student’s *t*-test is less than 0.5.

### ^1^H NMR experiments for monitoring CAR-HSC interactions in the presence of Ca ions

In order for the CAR-HSC complex to work well as an exfoliating agent, the breakup process of CAR-HSC interactions is needed to make new interactions between CAR and calcium (Ca) ions after penetration into the SC layers. For verification of the breakup process, we conducted a ^1^H NMR experiment for investigating the change of the CAR-HSC interaction on adding Ca ions. NMR is basically used to predict the structure of organic molecules, but recently, its application for investigating molecular interactions have been reported^29–31^. Zhao *et al*. showed the changes of NMR chemical shift caused by the chelation of peptide to calcium ions, which affects the electron density around the peptide protons. Kwamman *et al*. showed that the ^1^H NMR peak of a positively charged group of chitosan decreased below the saturation concentration by the restriction in motion caused by electrostatic interactions with negatively charged lysolecithin. These previous studies showed that NMR could be an alternative method for the evaluation of various interactions, including electrostatic or chelating interaction.

**Figure 6:**
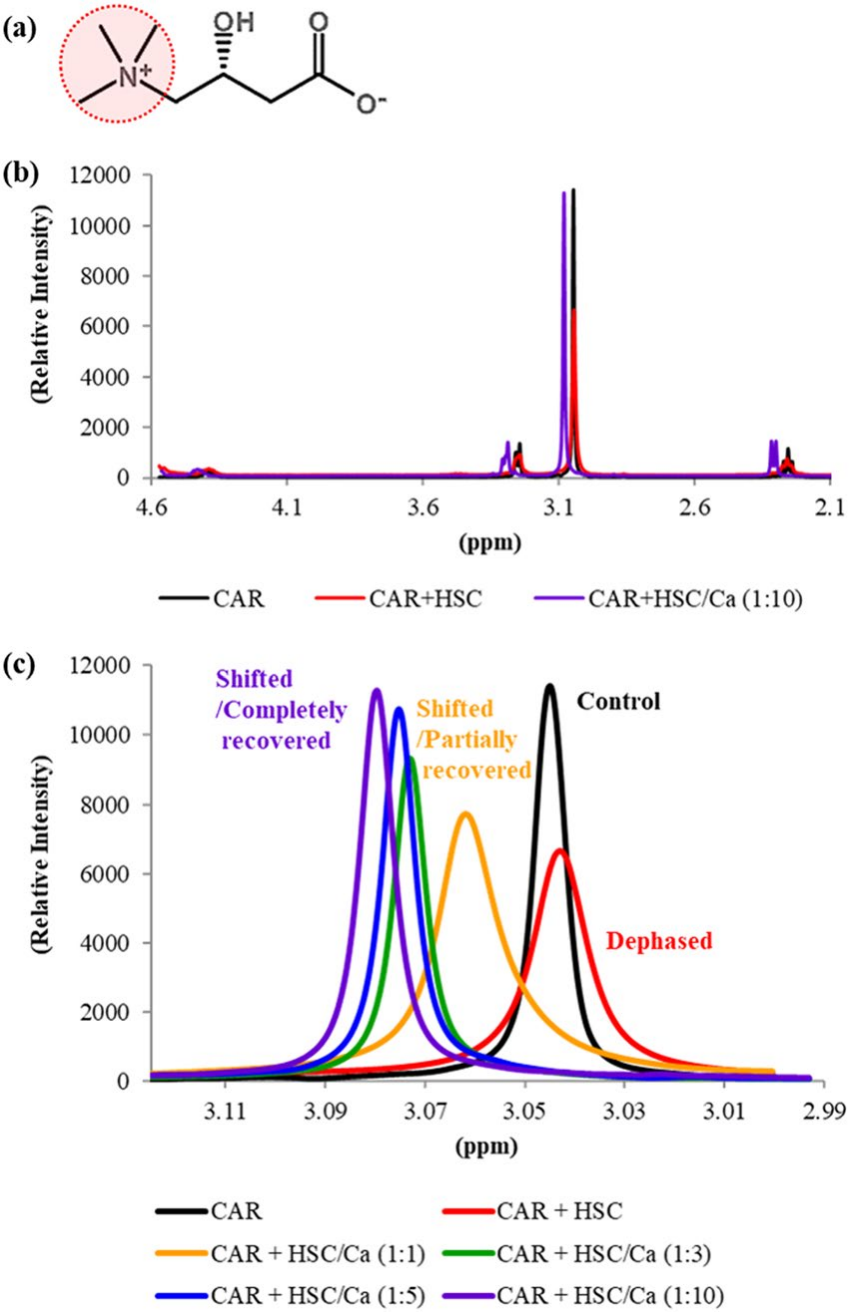
Dynamic NMR spectral changes of CAR. Molecular structure of CAR represented as dot line for its amino group (**a**). ^1^H NMR spectrum of CAR only and mixed with HSC in the presence of Ca ions or not (**b**). Alteration of ^1^H NMR spectra for amino group of CAR as mixed with HSC and adding Ca ions (**c**).

Dynamic alterations of NMR spectra for CAR applied with HSC and Ca ions are shown in Fig. 6. The positively charged amino groups (-NH3+) of CAR participate in electrostatic interaction with HSC, while they do not participate in chelation for Ca ions. The alteration of NMR spectra for CAR reflected these tendencies well. The NMR peak for amino groups in CAR decreased with HSC and gradually increased again on adding Ca ions. When the number of Ca ions is 10 times that of CAR molecules, the dephasing pulse for CAR was completely recovered (Fig. 4(a),(c)). These reductions in the NMR peak intensity were caused by a decrease in free amino groups on CAR due to the ionic interaction with HSC.

Moreover, we could find that the NMR spectra for each proton of CAR were overall sifted on introducing Ca ions (Fig. 4(b)). It is considered that these changes were caused by the chelation of CAR to Cal ions, which affects the electron density around the CAR protons. When the electron density decreased, the shielding effect weakened and the resonance frequency increased; consequently, the signal peaks moved to lower magnetic fields. These results imply not only that the CAR-HSC complex could completely breakup their interactions when Ca ions are more than 10 times the CAR molecules penetrating SC layers, but also that the dominant factor for such disruption is the affinity between CAR and Ca ions.

Our study showed that NMR is an effective method to investigate complex systems involving more than two molecules as well as their interactions. There are various classical methods, such as the measurement of electrical charge, zeta potential, or chelating power, to evaluate the interaction between two molecules. In these cases, NMR study may be an alternative method. On the other hand, classical methods that detect the changes of properties for the overall system cannot provide accurate information on what occurred among individual components in the complex system. Therefore, NMR spectroscopy is more useful for complex systems, providing information on changes in each proton scale with different types of signals appearing as different types of interaction.

### Effect of counter ions on the exfoliation efficacy of CAR

**Figure 7:**
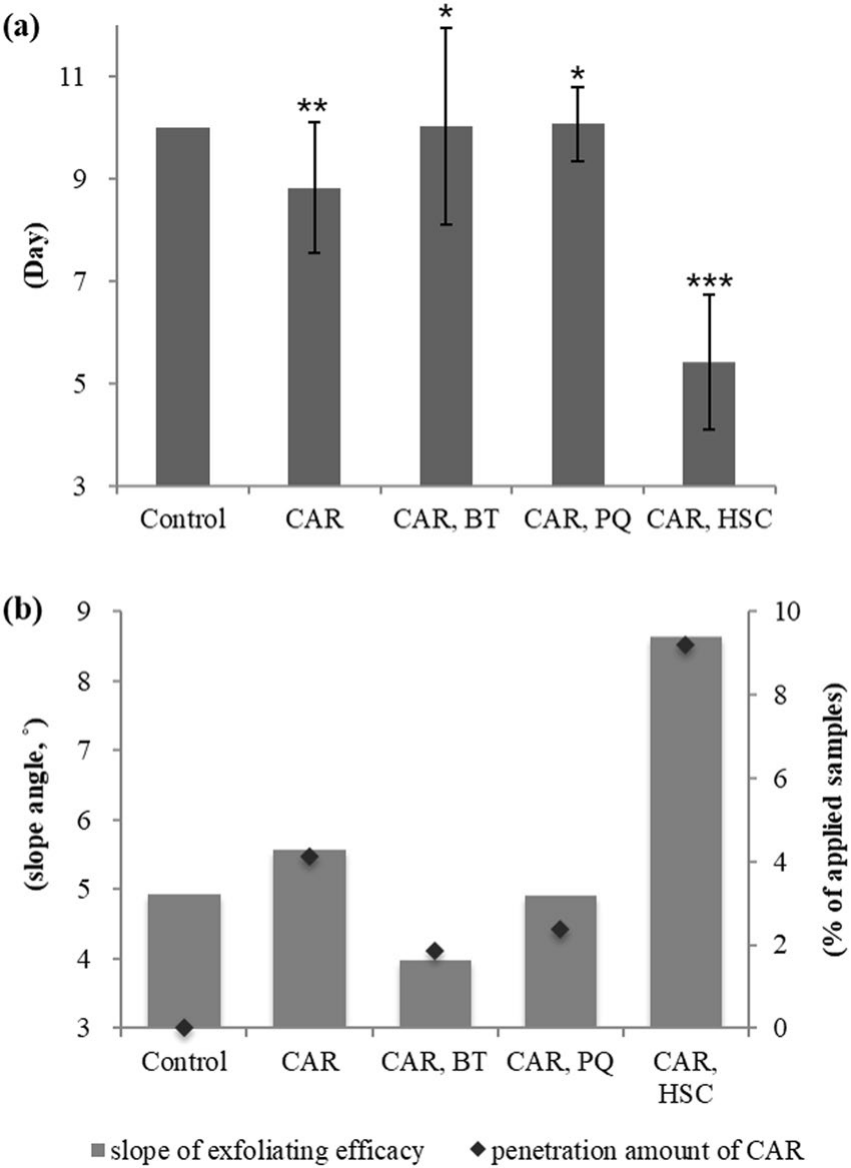
Exfoliation efficacy changes of CAR as mixed with BT, HQ, HSC. Exfoliation efficacy for each sample is represented as the SC turnover time for 50 percent SC exfoliation (**a**). Untreated case is used as negative control. The dose of CAR is 0.5 wt% and BT, HQ or HSC is mixed as 1:1 molar ratio with CAR. Data are means and bars represent the standard deviation. *P*-value for CAR is vs. negative control and for each mixture is vs. CAR only. *p < 0.05, **p < 0.01, ***p < 0.001; Student’s *t*-test. Correlation between the penetration amount of CAR and its exfoliating efficacy is represented in (**b**).

In this study, we carried out *in vivo* skin exfoliation tests using the DHA staining method to determine whether the change of the penetration property of CAR could improve exfoliation efficacy with 0.5 wt% CAR. The counter ions BT, HQ, or HSC, were mixed at 1:1 molar ratio with CAR. We set the untreated case as negative control. The calculated SC 50% turnover time (TT 50%) values are shown in Fig. 7. The result of the cases with only BT, HQ, or HSC applied, and the colour recovery images of DHA-stained SC of subjects are also represented in Supplementary Figs S1 and S2, respectively.

The tendency of the exfoliation efficacy of CAR decreasing with BT or HQ and increasing with HSC seems to be closely related with the penetration property in SC. This result suggested that the intensity of the exfoliating effects of CAR on skin depends to a large extent on its penetration amount in SC layers. Our study showed that introducing a zwitterionic amphiphilic substance to CAR is effective to enhance both percutaneous penetration and exfoliation efficacy.

Given the enhanced effect of CAR for SC exfoliation induced by HSC, further studies are required to investigate the therapeutic effect in real skin models. Clinical studies for skin conditional changes, such as skin roughness and lightness, with our exfoliating complex will be our focus in future work.

## Conclusions

In this work, we have studied the change of percutaneous penetration properties of CAR by the ion-pair method through *in vitro* and *in vivo* tests. The results show that the penetration ability of CAR could be significantly enhanced by the amphiphilic zwitterionic molecule, HSC. Moreover, the penetration depth of CAR has been verified in comparison with representative AHAs, glycolic acid and lactic acid. The experimental results suggest that this amino-acid-derived substance and its ion-pair complex could effectively act as an exfoliating agent, and the possibility of side effects related with deeper penetration is minimised. We also studied the alteration of the CAR-HSC ion complex induced by Ca ions by using ^1^H NMR experiments. This NMR study indicated that Ca ions could break up the ionic interaction between CAR and HSC to produce new interactions between CAR and Ca ions. This result not only enables us to determine how the CAR-HSC complex properly performs SC exfoliation after penetration in SC layers, but also implies that NMR can be a powerful tool for investigating interactions between molecules. Finally, the significantly enhanced exfoliation efficacy of CAR induced by HSC has been verified. Our study implies that such a formulation may be considered by commercial cosmetics companies or dermatologists to improve exfoliation and maintain healthy skin.

## Methods

Our study design and consent forms were approved by the institutional review board of LG Household and Health Care, Ltd. The institutional review board is operated independently including external evaluation members in accordance with the Korean Bioethics and Safety Act and certified by the Ministry of Health and Welfare of Korea. An independent data monitoring committee reviewed safety and interim efficacy analysis.

### Materials

L-Carnitine was purchased from Lonza (Switzerland). Hydrogenated soya phosphatidylcholine (HSC) was purchased from JJ-Dagussa (Thailand). Betaine (BT) was purchased from Asahi Kasei Chemicals Corporation (Japan). Polyquaternium-51 (PQ) was obtained from KCI (South Korea). PEG-40 stearate (PEGS) was purchased from CRODA (UK). Dihydroxy acetone (DHA) was purchased from MERCK (Germany). Calcium chloride and deuterium oxide (99 at%; D grade) were purchased from Sigma Chemical Company (USA). Phosphate-buffered saline (PBS) was purchased from GIBCO (USA).

Porcine skin (back skin; 1-mm thickness) was purchased from MediKinetics (South Korea) for the evaluation of skin penetration *in vitro*. A semi-occlusive sheet (Fixomull^®^) for skin tanning with DHA was purchased from 3 M Company (USA). Stripping tapes (D-Squame tape) and a pressure applicator (D-Squame pressure applicator) for collecting SC samples were purchased from CuDerm Corporation (USA).

### Preparation of samples

For water-soluble materials, each sample was prepared by simply dissolving in distilled water. For HSC, the sample was prepared by a sonication method, as described previously^32^. HSC was dispersed in distilled water at 85 °C, mixed for 30 min by using a homomixer (T. K. Robomix) (PRIMIX Corporation, Japan), and sonicated for 30 min by using a sonicator (Branson 2210R-DTH) (Branson, USA).

### *In vitro* penetration study: Franz diffusion cells

This procedure utilised the Franz cell method, which has been described previously^33,34^. For this investigation, modified Franz cell apparatus and porcine skin cut into 2.5 cm × 2.5 cm × 1 mm was used. The apparatus consisted of donor and receptor chambers. The area for diffusion was 254.3 mm^2^, and the receptor chamber was fully filled with 2.5–2.7 mL of PBS. After the sample was applied on the skin surface of the donor part, the cell was incubated at 37°C for 24 h. After the incubation, the skin surface was washed to remove unabsorbed samples.

The SC of porcine skin was collected by repeated stripping for 11 times with a pressure applicator and stripping tapes. The number of repetitions of tape stripping was determined as 11 based on previous studies^35,36^. Each tape piece was placed in a 1.5-mL test tube, mixed with 1 mL of distilled water, and shaken for 4 h. The shaken solutions for the 11 tapes were collected to determine the sample absorption in SC.

The remaining porcine skin was first soaked in distilled water at 80 °C for 30 s and then separated into epidermis and dermis layers by applying a slight pressure. Each collected layer was mixed with 1 mL of PBS solution, fragmentised using a tissue homogeniser (Precellys 24) (Bertin Instrument, France) for 1 min, and centrifuged (Centrifuge 5427R) (Eppendorf Company, Germany) at 13000 rpm for 5 min. Each supernatant and the receptor medium were collected to determine the amount of penetrated sample by using the liquid chromatography-ultraviolet (LC-UV) method.

### *In vivo* penetration study

Skin penetration can be assessed *in vivo* by using the standard stripped skin method^37^. Seven women aged 20–30 participated in the study after giving written informed consent, and the procedure followed the Declaration of Helsinki. Each sample was applied using a syringe on the inner lower arm three times every hour. The weight of syringe before and after the application was measured to check the amount of sample applied. The residue was rinsed off with distilled water. Subsequently, tape stripping for 11 times and sample analysis were conducted with the same method as in the previous penetration assay.

### *In vivo* evaluation of SC turnover time

SC turnover rates were evaluated using the DHA staining method^38^. Fifteen healthy volunteers aged 20–40 participated in the study after giving written informed consent, and the procedure followed the Declaration of Helsinki. About 0.4 mL of 10% DHA was applied onto the inner upper arms with a semi-occlusive sheet for 10 h. After 72 h, the sites of the brown area were assessed using a chromameter (CR-400, Konica Minolta, Japan), and the degree of discoloration with the sample applied twice a day was measured every day. A turnover time of 50% (TT 50%) refers to the time taken to replace 50% of the existing SC and thus recover the brown area by 50%. In this experiment, TT 50% was compared through a comparative analysis, and the results of the negative control group (untreated case) were calculated to be 10 days.

### Determination of conductivity

The ionic conductivity of each sample was measured at 25 °C by using a conductivity electrode (LE703, Mettler Toledo, USA) connected to a conductivity metre (FiveGo Cond meter F3, Mettler Toledo, USA) over a range of 0.01–200 mS/cm.

### ^1^H NMR spectroscopy

One-dimensional (1D) ^1^H NMR spectra were recorded at 25 °C on an NMR spectrometer (Bruker Ascend TM 500 NMR, Bruker Corporation, Germany) using standard NMR tubes with a diameter of 5 mm. The samples for the NMR study were prepared with the methods mentioned above by using deuterium oxide as a solvent.

### Statistical analysis

All data were expressed as mean ± standard deviation and analysed by the two-tailed paired t-test; * indicates p < 0.05, ** indicates p < 0.01, and *** indicates p < 0.001.

## Supplementary information

Supplementaty Information
