# Supplementary material for: Enhancement of exfoliating efficacy of L-carnitine with ion-pair method monitored by nuclear magnetic resonance spectroscopy
Source: Sci Rep. 2019 Sep 18;9:13507. doi: 10.1038/s41598-019-49818-2 (PMC6751292; doi:10.1038/s41598-019-49818-2)

**Supplementary Information for**

**Enhancement of exfoliating efficacy of L-carnitine with ion-pair method monitored by nuclear magnetic resonance spectroscopy**

**Sohyun In, Naeun Yook, Jin-Hyun Kim^+^, Munju Shin^+^, Suryeon Tak, Jeong Hoon Jeon, Byungjun Ahn, Sun-Gyoo Park, Cheon-Koo Lee, Nae-Gyu Kang***

R&D Campus, LG Household & Health Care, 10, Magokjungang 10-ro, Gangseo-gu, Seoul, Republic of Korea.

[^*^ngkang@lghnh.com](mailto:*ngkang@lghnh.com)

^+^These authors contributed equally to this work

**Supplementary Figure S1. Exfoliation efficacy of CAR when mixed with BT, HQ, and HSC. The cases of application of the counter ion only are also shown.** Exfoliation efficacy is represented as the SC turnover time for 50% SC exfoliation. Untreated case is used as negative control. The dose of CAR is 0.5 wt% and BT, HQ or HSC is used at 1:1 molar ratio with CAR. Data are means and bars represent the standard deviation. *P*-value for CAR is vs. negative control and for each mixtures is vs. CAR only. **p*<0.05, ***p*<0.01, ****p*<0.001; Student’s *t*-test.


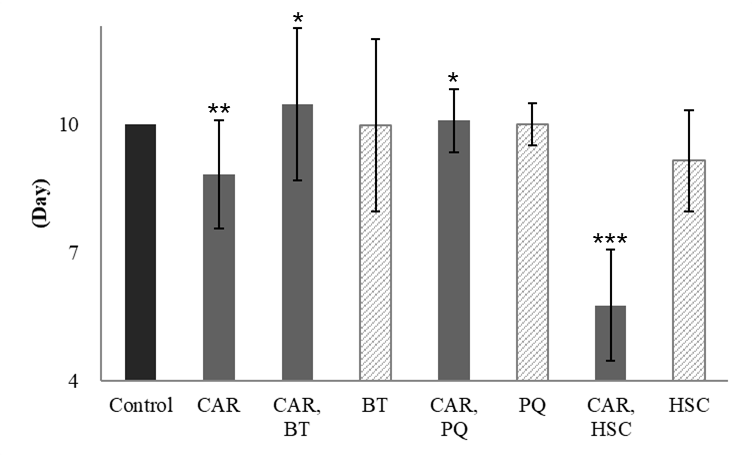


**Supplementary Figure S2. Color recovery of DHA-stained SC.** Photographs of the color recovery of DHA-stained SC taken from subject’s staining regions after 10 days. The dose of CAR is 0.5 wt% and BT, HQ, or HSC is mixed at 1:1 molar ratio with CAR. Color recovery of untreated (1), BT-treated (2), and CAR-treated (3) sites on one side of the arms and untreated (4), CAR-treated (5), and CAR and HSC-treated (6) sites on the other side of the arms (a). The case of CAR and HSC-treated (1), CAR-treated (2), HSC-treated (3), CAR and BT-treated (4) and untreated (5) sites (b,c). The case of CAR and HSC-treated (1), CAR-treated (2), CAR and BT-treated (3), CAR and HQ-treated (4) and untreated (5) sites (d,e).


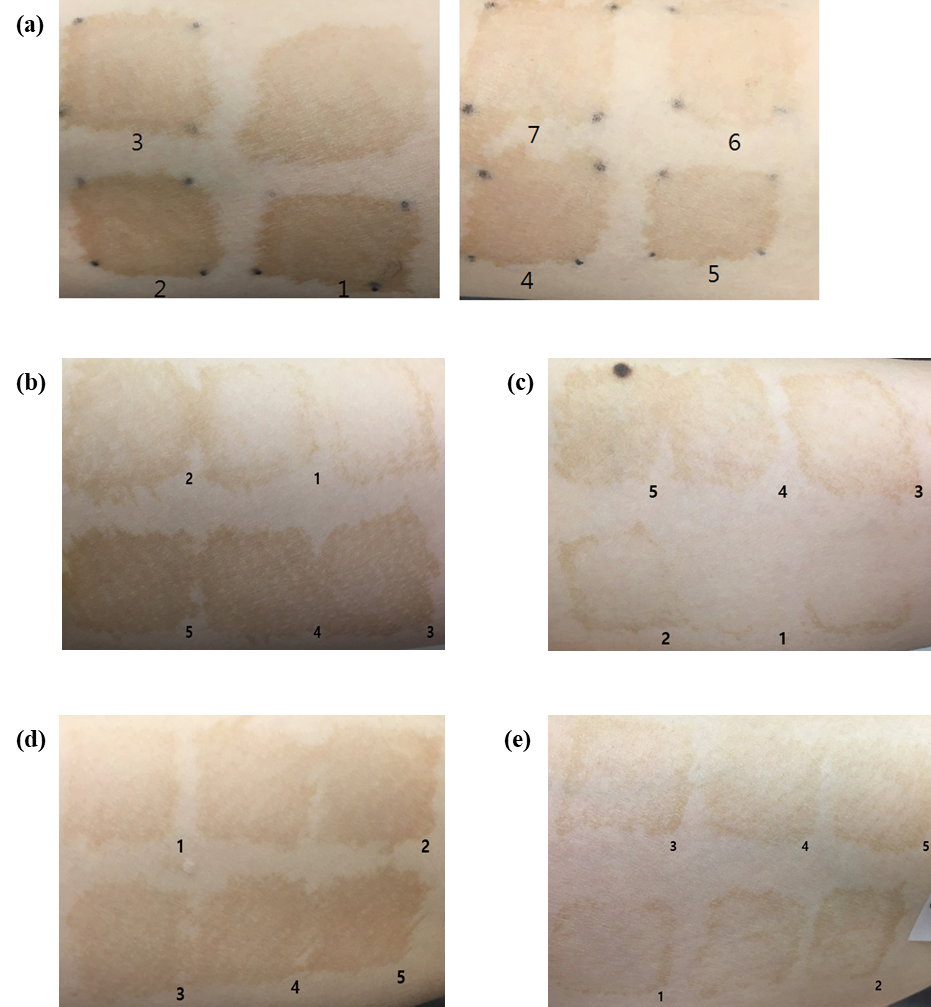

Supplement: Supplementary file 1 — Supplementaty Information [file 41598_2019_49818_MOESM1_ESM.docx]
